# Diagnoses-related procedure bundles in outpatient care – results from a research project using secondary data

**DOI:** 10.1186/1472-6963-11-S1-A3

**Published:** 2011-10-19

**Authors:** N Pfeffer, A Weisser, G Endel, C Scholler, A Eisl, P Filzmoser

**Affiliations:** 1 Main Association of the Austrian Social Insurance Institutions, Vienna, Austria; 2 University of Economics and Business, Vienna, Austria; 3 Vienna University of Technology, Vienna, Austria

## Introduction

Currently, one aspect of the discussion concerning healthcare reform in Austria focuses on strengthening the provision of ambulatory healthcare. Consequently, legal changes aim at fostering the development of new structures in healthcare (group practices), as well as implementing alternative payment mechanisms for those entities.

In 2009, we started a research project in the field of diagnosis-related mechanisms of payment for ambulatory care. The project focuses on episodes of care for chronic diseases. The main objectives of this project are to show the feasibility of the available administrative healthcare data, to develop a statistical toolkit in order to identify diagnoses-related procedure bundles in ambulatory care, and to calculate costs for the procedure bundles.

## Methods

We use a pseudonymous dataset that contains a full record of ambulatory health data as well as hospital data for 2006–2007. The data is linked using a unique patient identifier.

When calculating procedure bundles, only costly procedures from outpatient care were included. Therefore, we used descriptive statistics to identify the relevant procedures for each specialty. Diagnoses were obtained from ATC-Codes of prescription data and were assigned to each patient via his or her personal record of medication. We limited our research to a number of common chronic diseases (e.g., diabetes, COPD/asthma, dementia).

Three different approaches were used to include patients in the data sample:

1. Patients with no other disease than the disease in question for the time t0 +/- 6 months

2. Patients with no other disease than the disease in question for the time t0 +/- 1 month

3. All patients having the disease in question for the time t0 regardless of any other disease

Next, we applied linear regression to identify those procedures that are significantly related to the single diagnoses included in the sample (within a time span of -90 days/ +180 days from the diagnosis). The significance was measured by the frequency of a particular procedure with respect to all diagnoses of a disease included in the sample.

Finally, we defined procedure bundles as all procedures to the left of the most significant difference between two adjacent procedures.

## Results

Results show that for most of the diseases we considered procedure bundles can be identified using the methods described above. To a large extent, significant procedures for each diagnosis represent technical procedures, such as determining laboratory values or ECGs. In addition, expected non-technical procedures (e.g., eye treatment for diabetes patients) could be allocated to the relevant diagnoses. However, we were unable to find feasible bundles for diagnoses where “quasi-unique” ATC-Codes do not exist. The bundles did not vary substantially across the three methods used to include diagnoses in the sample.

## Conclusions

The results obtained can be seen as the first step towards describing procedure bundles related to a number of chronic diseases. In the next step, experts will need to refine the bundles. These bundles provide a solid ground for the calculation of costs for diagnosis-related procedure bundles in ambulatory care. The methods used can be implemented in other data sets as well, and are therefore not limited to the context of the Austrian healthcare system.

